# A Shared Decision-making Tool for Drug Interactions Between Warfarin and Nonsteroidal Anti-inflammatory Drugs: Design and Usability Study

**DOI:** 10.2196/28618

**Published:** 2021-10-26

**Authors:** Thomas J Reese, Guilherme Del Fiol, Keaton Morgan, Jason T Hurwitz, Kensaku Kawamoto, Ainhoa Gomez-Lumbreras, Mary L Brown, Henrik Thiess, Sara R Vazquez, Scott D Nelson, Richard Boyce, Daniel Malone

**Affiliations:** 1 Vanderbilt University Nashville, TN United States; 2 University of Utah Salt Lake City, UT United States; 3 University of Arizona Tuscon, AZ United States; 4 University of Heidelberg Heidelberg Germany; 5 Department of Biomedical Informatics University of Pittsburgh Pittsburg, PA United States

**Keywords:** shared decision-making, user-centered design, drug interaction, clinical decision support

## Abstract

**Background:**

Exposure to life-threatening drug-drug interactions (DDIs) occurs despite the widespread use of clinical decision support. The DDI between warfarin and nonsteroidal anti-inflammatory drugs is common and potentially life-threatening. Patients can play a substantial role in preventing harm from DDIs; however, the current model for DDI decision-making is clinician centric.

**Objective:**

This study aims to design and study the usability of DDInteract, a tool to support shared decision-making (SDM) between a patient and provider for the DDI between warfarin and nonsteroidal anti-inflammatory drugs.

**Methods:**

We used an SDM framework and user-centered design methods to guide the design and usability of DDInteract—an SDM electronic health record app to prevent harm from clinically significant DDIs. The design involved iterative prototypes, qualitative feedback from stakeholders, and a heuristic evaluation. The usability evaluation included patients and clinicians. Patients participated in a simulated SDM discussion using clinical vignettes. Clinicians were asked to complete eight tasks using DDInteract and to assess the tool using a survey adapted from the System Usability Scale.

**Results:**

The designed DDInteract prototype includes the following features: a patient-specific risk profile, dynamic risk icon array, patient education section, and treatment decision tree. A total of 4 patients and 11 clinicians participated in the usability study. After an SDM session where patients and clinicians review the tool concurrently, patients generally favored pain treatments with less risk of gastrointestinal bleeding. Clinicians successfully completed the tasks with a mean of 144 (SD 74) seconds and rated the usability of DDInteract as 4.32 (SD 0.52) of 5.

**Conclusions:**

This study expands the use of SDM to DDIs. The next steps are to determine if DDInteract can improve shared decision-making quality and to implement it across health systems using interoperable technology.

## Introduction

### Background

Drug-drug interactions (DDIs) are preventable adverse events that are responsible for 5% to 14% of adverse drug reactions in patients that are hospitalized [1,2], are a major risk factor for hospitalization [3], and occur in up to 13% of older adult ambulatory patients [4-6]. Exposure to life-threatening DDIs occurs despite the widespread use of clinical decision support. Alarmingly, up to 24% of patients on warfarin receive a prescription for a nonsteroidal anti-inflammatory drug (NSAID), which increases the risk of gastrointestinal bleeding up to twofold [7,8].

Most electronic health records (EHRs) implement DDI clinical decision support functionality with underlying logic provided by drug knowledge base vendors, but DDI alerts continue to be overridden at rates as high as 90% [9-11]. The current model for DDI decision-making is highly clinician-centric in spite of the fact that patients can play a substantial role in preventing potential harm due to DDIs. Studies that have explored different clinical decision support for DDIs [12,13] indicate that interactive decision dashboards have the potential to foster informed decision-making by patients [13]. These decision aids allow patients and clinicians to deliberate together about the advantages and disadvantages of different therapies and arrive at decisions that are concordant with the best available evidence, clinician knowledge, and patient preferences [14,15].

Accordingly, the overarching goal of this study is to incorporate patient-centered shared decision-making (SDM) for addressing DDIs, an advance from clinician-centric decision-making models. SDM is a conversation where patients share their values and preferences to choose a treatment that aligns with their goals [16,17]. Electronic decision aids can support this conversation; however, SDM is uncharted in the DDI domain [18].

### Objectives

The purpose of this study was to design and evaluate the usability of DDInteract, an SDM tool for the warfarin and NSAID DDI.

## Methods

### Overview

**Figure 1:**
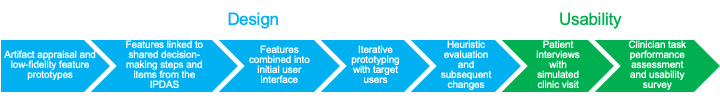
Summary of the design and usability process for DDInteract. IPDAS: International Patient Decision Aid Standards Collaboration.

The design and usability assessment of DDInteract was guided by user-centered design principles and an SDM framework. The user-centered process included iterative and overlapping steps of prototyping (ie, low fidelity, stable, and high fidelity), stakeholder feedback, and usability heuristics and testing [19-24]. The SDM framework consists of five steps: (1) seek your patient’s participation, (2) help your patient explore and compare treatment options, (3) assess your patient’s values and preferences, (4) reach a decision with your patient, and (5) evaluate your patient’s decision [25]. Figure 1 depicts a summary of the design and usability process. This study was approved by the University of Utah Institutional Review Board.

### Design

The design team consisted of multidisciplinary experts in DDIs, clinical decision support, patient and provider communication, SDM, and pharmacotherapy outcomes. The process began with an artifact appraisal of SDM tools, DDI alerts, and clinical practice materials relevant to anticoagulants. Information from this appraisal was used to sketch low-fidelity feature prototypes, which were reviewed and discussed with the design team in weekly meetings. Features and functionality deemed important by the design team were retained for future iterations. Features were linked to the SDM steps and checklist items from the International Patient Decision Aid Standards Collaboration [26]. Once the design team coalesced on preliminary feature designs, these were combined into an initial protype of the complete user interface using Adobe XD (Adobe Inc).

Target users (ie, 2 physicians and 1 pharmacist) were individually shown the initial complete user interface prototype and asked to provide feedback on the usefulness, aesthetics, proposed functionality, and content. Several iterations were made in collaboration with these target users until no substantial feedback was provided. At this point the prototype was considered stable enough for a heuristic evaluation. The heuristic evaluation was based on knowledge of Nielsen's 10 Usability Heuristics for User Interface Design [27,28] and was performed by two experts with training and experience in human-centered design, psychology, and medical informatics. The goal of the heuristic evaluation was to identify design flaws that could be addressed prior to conducting resource-intensive testing. Specific feedback regarding design that might impede users’ goals were noted and shared in a team meeting along with a discussion of potential solutions for each flaw. Once the stable prototype was modified to address findings from the heuristic evaluation, it was considered high fidelity and ready for usability testing.

### Usability

Usability assessments consisted of two parts: (1) patient interviews with simulated clinic visits and (2) clinician task performance assessments and usability surveys. DDInteract was designed to be used by clinicians at the point of care. Since patients would not use DDInteract without a clinician present, we did not test task completion success and efficiency with patients.

#### Patients

Patient participants were recruited from the anticoagulation service at the University of Utah. Participants were required to be on warfarin for a chronic condition such as atrial fibrillation. The perceived usability and usefulness of DDInteract was assessed with participants individually through two simulated clinical scenarios and a semistructured interview. Participants were given two short clinical vignettes to read before the session (Multimedia Appendix 1, Table S1). The decision associated with each vignette was whether to start an NSAID for pain. The vignettes were designed to test the range of responses based on a patient’s risk (ie, high risk and low risk) of gastrointestinal bleeding. In the high-risk vignette, the patient had multiple risk factors for gastrointestinal bleeding including age older than 65 years, use of an antidepressant, and history of a gastrointestinal bleeding. In the low-risk vignette, warfarin was the only risk factor. Participants simulated SDM based on the clinical vignettes with a provider (author KM). Following the clinical scenarios, patients were asked questions pertaining to aspects of DDInteract, the use of DDInteract for SDM, and the utility of SDM for DDIs. The interviews were conducted online with audio and screen recording. The audio was transcribed and coded into general topics.

#### Clinicians

Physicians and pharmacists with anticoagulation therapy experience were recruited by snowball sampling. The overarching goal of the clinician usability assessment was to obtain objective and subjective data on the use of DDInteract. Participants were asked to complete a task performance assessment and a perceived usefulness survey. Participant characteristics were collected as part of the survey. Links to the instructional video, task performance assessment, and survey were emailed to participants. The instructional video was a brief introduction to DDInteract. The task performance assessment was web-based and recorded the participant’s screen. The survey was based on the System Usability Scale and included a free-text section for feedback [29,30]. Tasks consisted of eight key navigation and functionality tasks (Table 1). Performance was measured by task completion rates and the time to complete each task. After a task was completed, the app reset to the home screen. The time was measured from when the home screen was displayed to when the task was completed.

**Table 1:** Clinician task prompts and actions performed that result in successful completion.

Tasks	Success
1. Your patient has questions about what a gastrointestinal bleed is. Please navigate to patient education about a gastrointestinal bleeding.	Navigating to and clicking on the drop-down arrow for “What is a gastrointestinal (Stomach) bleed?”
2. With previous patients, you have found it confusing for them to understand the drug class NSAIDs^a^. Please find the picture of multiple NSAIDs to illustrate how not only ibuprofen is an NSAID.	Navigating through the “What is a drug-drug interaction” drop-down and clicking on the “NSAID” hyperlink
3. Your patient informed you that they stopped taking fluoxetine. Please remove fluoxetine (Prozac) as a risk factor to show how their risk has changed.	In the patient Risk Profile section, the toggle for “On Selective Serotonin Reuptake Inhibitor” was preconfigured in the on position. The successful action was clicking the toggle off.
4. Assume your patient would like to take a medication then click the button to view medication options.	Navigating to the decision tree questions and clicking on the “Medication” button
5. Your patient has decided to try non-NSAID medication options. Please select acetaminophen (Tylenol) and lidocaine (Lidoderm).	Navigating to the second question of the decision tree and clicking “Other medications” then selecting “acetaminophen (Tylenol) 500mg” and “lidocaine (Lidoderm) 5% patch”
6. Your patient believes NSAIDs help the most with pain but would like to reduce their risk. Please select the oral NSAID option with the least gastrointestinal bleed risk. Then select that a stomach acid reducer is not needed.	Navigating to the second question of the decision tree and clicking on “Oral NSAID” then selecting “celecoxib”
7. Your patient insists on taking medications only once per day. Please select the oral NSAID option with the most risk and add esomeprazole (Nexium).	Navigating to the second question of the decision tree and clicking on “Oral NSAID” and selecting “meloxicam.” Then clicking on “Stomach acid reducer” and selecting “esomeprazole.”
8. Please place any order in the queue for one of the treatment options.	Navigating through the decision tree and clicking “Accept”

^a^NSAID: nonsteroidal anti-inflammatory drug.

## Results

### Design

Although DDIs are a novel application for SDM, we did assess several relevant electronic decision aids such as those used for cardiovascular and diabetes management [31]. We compared a variety of relevant decision aids to the SDM steps and the International Patient Decision Aid Standards checklist [26]. Generally, decision aids lacked features to elicit patient values and preferences. Additionally, decision aids varied on the content provided to make treatment decisions including price, effectiveness, and side effects of treatment [32].

**Figure 2:**
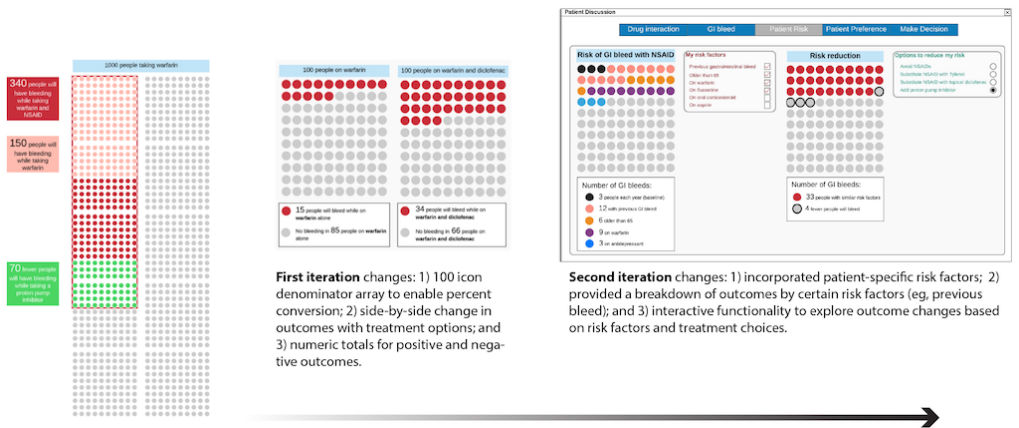
Icon array evolution, from left to right, through two iterations. NSAID: nonsteroidal anti-inflammatory drug.

**Figure 3:**
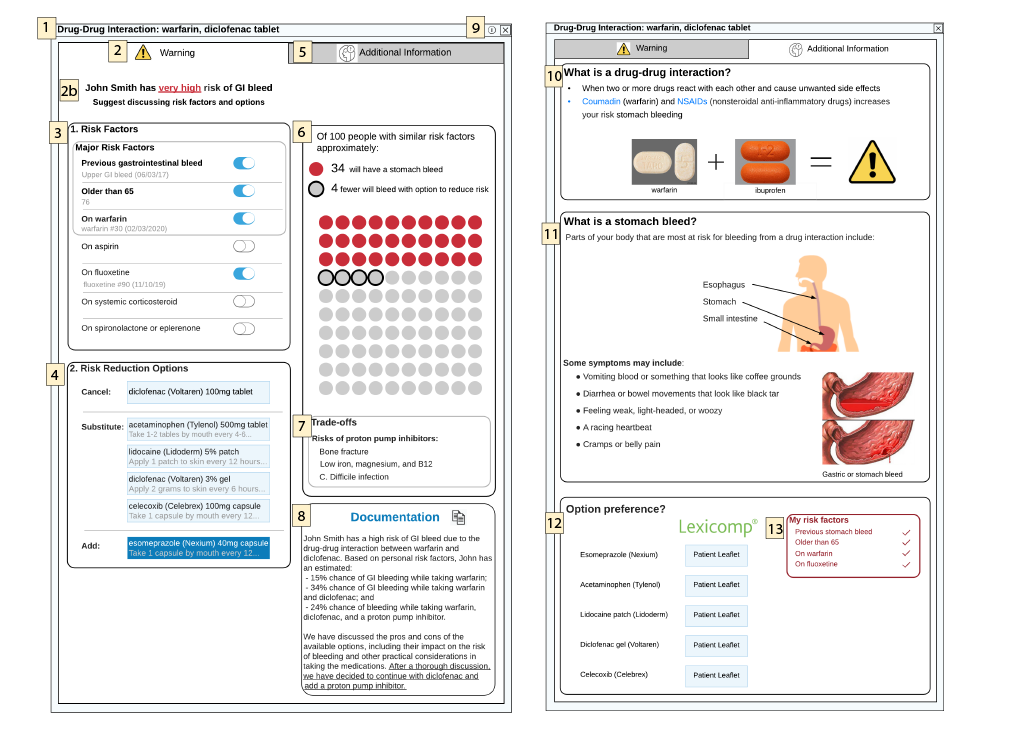
Stable prototype used for heuristic evaluation. Numbers refer to items described in the heuristic report. GI: gastrointestinal.

Several features from relevant decision aids were adapted to the DDI use case. Features included the icon array, personalized risk, and ability to simulate risk based on patient factors and treatments. Features and functionality evolved through multiple iterations. For example, icon arrays have shown promise in communicating risk to patients and clinicians [33-35]. Figure 2 depicts how the icon array changed over two iterations. Icon array features were based on findings from the literature and expert feedback [36,37]. Once features such as the icon array were acceptable from the design team’s perspective, they were adapted and placed into the complete user interface (ie, stable prototype). Figure 3 depicts the stable prototype used for the heuristic evaluation. Overall, 2 major, 13 moderate, and 14 minor issues were identified. One of the two major issues was associated with the number four in Figure 3. The evaluators thought that users may not correctly interpret “Substitute” and “Add” when selecting treatment options. In response, we removed the order entry context, which will avoid a user from referencing the warfarin or NSAID order in process.

**Figure 4:**
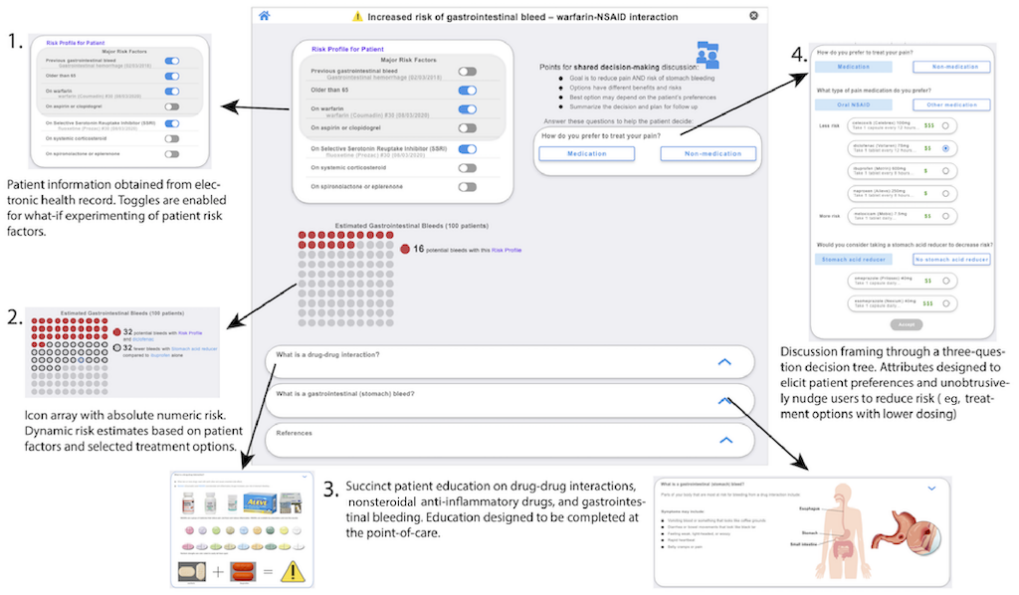
Final DDInteract high-fidelity prototype used in the usability study along with feature description. NSAID: nonsteroidal anti-inflammatory drug.

The DDInteract prototype used for evaluating usability consisted of four sections. The first section in the top left corner of Figure 4 is a patient-specific risk profile. Substantiated risk factors for gastrointestinal bleeding are listed and stratified by risk and supporting evidence. When patient-specific risk factors are pulled from the EHR, the toggle is on (ie, blue). Toggles can be manually changed to account for data not in the EHR or for clinicians to test different scenarios such as adding an antidepressant. The second section in the bottom left is a dynamic risk icon array. The icon array changes with risk factors and treatment options. Absolute numeric risk is provided along the visualization. The third section on the bottom of Figure 4 provides patient education and supporting evidence. Succinct and image-oriented patient education is provided for DDIs and gastrointestinal bleeding. Since risk factors and estimates are evidence based, information is provided on how these aspects were derived. The fourth section is a decision tree with three questions that help structure the conversation and support treatment decision-making. The questions were created and validated by clinicians with the aim to streamline the SDM process in the context of patient care appointments. Question attributes were designed to elicit patient values and preferences associated with bleeding risk and pain treatment. Unobtrusive nudges to reduce risk were used through prepopulated NSAID dosing and default proton pump inhibitor (PPI) selection when an NSAID is chosen. Finally, functionality for generating documentation of the SDM discussion was provided.

### Usability

#### Patients

All 4 participants were aged 65-85 years and had taken warfarin for more than 5 years. A provider (KM) and a facilitator (author TJR) interviewed each patient for approximately 1 hour. For the high-risk vignette (patient with multiple risk factors), all participants chose a combination of nonmedication treatment (eg, physical therapy) and acetaminophen. For the low-risk scenario (patient with minimal risk factors), most participants chose a short course of celecoxib or ibuprofen, with a PPI.

Although participant knowledge about the warfarin-NSAID DDI varied, all participants appreciated the ability to see DDInteract while the provider discussed risk and treatment options. One participant stated:

If I wasn’t able to see the [treatment] options, I wouldn’t know what to ask

Furthermore, participants felt empowered to participate in making decisions that aligned with their preferences by referring to the decision aid during discussion:

I personally don’t like taking medications and want two avoid taking more. It looks like I can try other ways to relieve my pain and I would prefer trying those

Participants wanted to have access to the decision aid or a printout, outside the encounter, to review what was discussed and decided. One participant stated:

I usually forget what we [patient and provider] talk about during the appointment, so I would go to my After Visit Summary to review what we talked about.

Most participants believed SDM was novel and different from past decision-making experiences with providers:

Doctors usually make decisions like these for me.

Two patients were unaware that the NSAID class included more than ibuprofen. Generally, participants valued SDM and using DDInteract with the provider. Furthermore, participants preferred to avoid additional medications and wanted to reduce the risk of gastrointestinal bleeding as much as possible.

#### Clinicians

A total of 11 clinicians participated in the usability evaluation (Table 2). Of the 11 participants, 3 stopped the task study after the first task. Of those 3 participants, 2 were pulled to clinical duties. The other participant failed to complete the second task and chose to stop the study rather than skipping the task. Of the 8 participants who completed the study, all were successful on each task. The mean time to complete eight tasks was 144 (SD 74) seconds. Table 3 delineates the task prompt and the mean time for completion. Screen capture was used to determine how participants navigated through the tool. A total of 11 participants completed the usability and satisfaction survey, with an overall mean rating of 4.32 (SD 0.52) of 5. Table 4 delineates mean ratings for each survey item.

**Table 2:** Participant characteristics for the usability evaluation.

	Participants, n	Specialty (n participants in each group)	Participant years of experience, n					Participant clinical percent effort, n					Self-assessed experience with warfarin from 0 to 100, mean (range)
			<5	6-10	11-15	>16	<21		21-40	61-80	>80		
Physicians	7	Family medicine (4), emergency/critical care (2), hematology (1)	2	2	2	1	0		2	3	2	67 (29-88)	
Pharmacists	4	Anticoagulation/ambulatory care (3), general (1)	0	0	2	2	1		0	1	2	93 (87-100)	

**Table 3:** Mean and SD for task time in seconds across usability participants (n=8).

Tasks	Mean time in seconds (SD)
1. Your patient has questions about what a gastrointestinal bleed is. Please navigate to patient education about a gastrointestinal bleeding.	39 (48)
2. With previous patients, you have found it confusing for them to understand the drug class NSAIDs^a^. Please find the picture of multiple NSAIDs to illustrate how not only ibuprofen is an NSAID.	42 (34)
3. Your patient informed you that they stopped taking fluoxetine. Please remove fluoxetine (Prozac) as a risk factor to show how their risk has changed.	2 (1)
4. Assume your patient would like to take a medication then click the button to view medication options.	3 (4)
5. Your patient has decided to try non-NSAID medication options. Please select acetaminophen (Tylenol) and lidocaine (Lidoderm).	30 (48)
6. Your patient believes NSAIDs help the most with pain but would like to reduce their risk. Please select the oral NSAID option with the least gastrointestinal bleed risk. Then select that a stomach acid reducer is not needed.	32 (35)
7. Your patient insists on taking medications only once per day. Please select the oral NSAID option with the most risk and add esomeprazole (Nexium).	13 (4)
8. Please place any order in the queue for one of the treatment options.	15 (10)

^a^NSAID: nonsteroidal anti-inflammatory drug.

**Table 4:** Clinician usability survey items and responses (n=11). Responses were on a 1 to 5 Likert scale where 1 is strongly disagree and 5 is strongly agree.

Survey items	Mean (SD)
I found the decision tool to be logical.	4.36 (0.67)
I found the decision tool to be efficient.	4.18 (0.75)
The decision tool was effective in the decision-making process.	4.36 (0.67)
The shared decision-making was valuable.	4.27 (0.79)
The decision tool was valuable.	4.36 (0.67)
I thought the decision tool was easy to use.	4.27 (0.65)
I enjoyed the experience.	4.36 (0.81)
I learned something new from this experience.	4.36 (0.67)

Clinician participants provided a variety of comments on the purpose and usefulness of DDInteract after completing the survey. Two participants thought the app would be helpful for patient education:

I think half the time they [patients] just think we’re [clinicians] being mean by telling them they shouldn’t take their NSAIDs. And the visual for how that can be mitigated is great. I actually think that the educational section of the tool would be helpful when we’re doing new educations for warfarin/DOACs [direct oral anticoagulants] even if we’re not doing a shared decision-making type thing.

Have you considered using this as a tool not just in the clinical setting but in the medical education setting?

Two participants had questions on where and when the app would be used:

Is this an app that will be on the provider’s phone or the intention is for the patient to download this app and fill it out themselves? Or will this be a website that is pulled up during an office visit where both parties are present in the room?

I'm not completely clear on the exact clinical situation in which this tool would be used and the point in the workflow in which that would happen.

One participant thought the tool could be expanded with a general guide on interpreting risk of gastrointestinal bleeding:

I know it is individualized, but I like how there are some general guidelines with the HAS-BLED score. It would be nice to have something similar. Also, at what risk is a PPI strongly recommended.

Finally, participants thought the dynamic risk calculation would be a feature they would return to the tool to use. The ability to toggle between risk factors and treatment options helped to quantify risk and explore different treatment options. Generally, participants believed DDInteract was easy to use and would support SDM.

#### Prototype Changes

Key changes were made in response to the patient interviews. Changes included expanding nonmedication and non-NSAID treatment options, adding functionality for selecting more than one non-NSAID treatment (eg, physical therapy and acetaminophen), and creating a printable handout and an after visit summary that patients can access outside the encounter. Based on the duration to complete certain tasks and how clinicians navigated through DDInteract, we made feature changes regarding tasks one and two (Table 3). The modifications included enabling the user to see the entire app without scrolling to the drop-down items for patient education and automatically displaying the images.

## Discussion

### Principal Findings

This study designed and assessed the usability of a tool for SDM with DDIs (Figure 4). Overall, it appears that SDM can be enhanced by using a tool that displays risks of harm and alternatives. The process of designing DDInteract was rigorous, applying user-centered design principles through iterative prototyping (Figure 2). Target users found DDInteract easy to use and believed it could be useful for supporting SDM (Tables 3 and 4). Given that DDI clinical decision support has been traditionally clinician centric, this study may contribute to a major shift in the way certain medication alerts are developed and used. Through the process designing and evaluating DDInteract with clinicians and patients, lessons were learned regarding patient decision-making and their understanding of the warfarin-NSAID DDI. Furthermore, lessons were learned from clinicians about when in the workflow DDI alerts are addressed and implementing SDM in routine patient care.

Not all DDIs are amenable to SDM, and clinicians should use their judgement before opening a discussion about certain DDIs. Although patients appreciated discussing the warfarin-NSAID DDI with the provider, only the low-risk scenario seemed to be relevant. Certain high-risk DDIs should be avoided without SDM. Additionally, although these patients had been on warfarin for multiple years, knowledge about DDIs and treating pain while taking warfarin was limited. This aligns with what others have found on patient knowledge about anticoagulant therapy [38,39]. Knowledge about which medications are NSAIDs and symptoms of bleeding should not be assumed despite experience with warfarin. Regardless of the decision to avoid an NSAID, patient education about the DDI is needed. Finally, patients mentioned that after previous provider encounters it was difficult to recall information about treatment decisions. Consideration for allowing patients to reference the tool after an encounter may help with comprehension and adhering to decisions; however, DDInteract and other similar tools would need to be adapted to and tested with patients to understand decision-making without provider assistance.

Clinicians had questions about when and how DDInteract would be used. Medication prescribing often occurs at the end of an encounter without the patient. If DDInteract was triggered when an NSAID is ordered, the patient might not be available for discussion. Opportunities to use an SDM tool for DDIs earlier in the workflow may be needed. For example, triggering DDInteract for a patient who is having pain or starting warfarin are additional use cases. Triggering on pain is especially relevant for patients on warfarin due to frequent use of over-the-counter NSAIDs. Although DDInteract was designed to mitigate risk associated with DDIs, clinicians requested decision support for other aspects of anticoagulant therapy, such as deciding to start an anticoagulant or which anticoagulant to use. To achieve broad uptake of SDM for DDIs, the scope of DDInteract may need to be expanded to other decision-making and clinician workflow opportunities.

### Future Research

The next step is to conduct a formative evaluation of DDInteract to understand how it impacts measures of decision-making, satisfaction, and clinician workflow. To maximize dissemination and enable integration with EHR systems, we have developed an interoperable DDInteract app using emerging clinical decision support standards including Clinical Quality Language, Clinical Decision Support Hooks, and SMART on Fast Healthcare Interoperability Resources (FHIR) [40]. A SMART on FHIR prototype of DDInteract is available on Logica Sandbox, and we have successfully implemented DDInteract in an EHR test environment at the University of Utah. Further research is needed to understand how SDM with DDIs can be integrated with overarching decisions surrounding anticoagulant therapy. Finally, research is needed to explore how decision aids in the EHR can be adapted to clinical workflows to enable SDM in routine patient care.

### Conclusion

This study describes the design and usability testing of DDInteract. The findings contribute to knowledge about implementing SDM in routine patient care and expand the use of SDM to DDIs. A multidisciplinary design team collaborated with patients, clinicians, and health information technology experts to design a tool that provides a patient-specific risk calculation, elicits patient preferences, and guides both the patient and clinician to a decision. The rigorous design process resulted in a usable and potentially useful tool. Through the design process and usability testing, key lessons were learned from the patient and clinician perspectives. The next step is to evaluate the utility of DDInteract in a clinical setting, and if successful, to implement it across EHRs using interoperable technology.

## Appendix

Multimedia Appendix 1 Clinic vignettes used for patient interviews.

## Abbreviations

DDI: drug-drug interaction
DOAC: direct oral anticoagulant
EHR: electronic health record
FHIR: Fast Healthcare Interoperability Resources
NSAID: nonsteroidal anti-inflammatory drug
PPI: proton pump inhibitor
SDM: shared decision-making
