# Supplementary material for: A Shared Decision-making Tool for Drug Interactions Between Warfarin and Nonsteroidal Anti-inflammatory Drugs: Design and Usability Study
Source: JMIR Hum Factors. 2021 Oct 26;8(4):e28618. doi: 10.2196/28618 (PMC8579222; doi:10.2196/28618)
Supplement: Multimedia Appendix 1 [file humanfactors_v8i4e28618_app1.docx]

**Appendix**

| **Scenario 1 (low-risk gastrointestinal bleeding)** | You are 55 years old and take warfarin every day for a heart condition and have knee pain. You made an appointment with your primary care doctor to see if there is anything you can do to reduce the knee pain.  The pain started a few years ago and has been getting increasingly worse. A previous doctor diagnosed the knee pain as osteoarthritis due to an injury when you were younger. The pain only happens when you are walking and it doesn’t require you to use any sort of walking assistance, such as a cane or walker. It hasn’t limited your ability to do daily tasks, but it does make your morning walk painful.  You have tried a couple over-the-counter treatments without much relief. First, you tried Bengay topical cream, which you applied after your morning walks. It didn’t seem to help. Second, you tried Tylenol (500mg). It seemed to only help a little when you took it before your walk. Third, you tried ibuprofen and it seemed to help but a friend told you not to take ibuprofen with warfarin, so you stopped taking it.  Since you try to be active, you would like to use a medication that will help you keep walking and working without so much pain. You ask the doctor to prescribe you a stronger ibuprofen. |
| --- | --- |
| **Scenario 2 (high-risk for gastrointestinal bleeding)** | You are 68 years old and broke your arm about three weeks ago when you tripped on the stairs. You made an appointment with your primary care doctor to see if there is anything you can do to reduce the pain in your broken arm.  Every day, you take warfarin for a heart condition and fluoxetine for anxiety and depression. In recent years, your gastric reflux (stomach acid) has gotten worse. A couple years ago, you were admitted to the hospital for a bleeding stomach ulcer. Since the hospitalization, you take Zantac (*make sure they know what it is*) a couple times a week when your stomach is upset but have not had another ulcer.  The first week after you broke your arm, the emergency room doctor prescribed Percocet (oxycodone 5mg and acetaminophen 325mg – 1 tablet every 4 hours) for the pain. This medication made you drowsy and constipated but did reduce the pain a lot. After the Percocet, you have been taking Tylenol (500mg twice daily) without much relief.  You would like to reduce the pain in your arm but would like to avoid taking Percocet if possible. You ask the doctor if you can take ibuprofen instead of Tylenol. |

Appendix, Table S1. Clinic vignettes used for patient interviews
